# Diffusion Kurtosis MR Imaging versus Conventional Diffusion-Weighted Imaging for Distinguishing Hepatocellular Carcinoma from Benign Hepatic Nodules

**DOI:** 10.1155/2019/2030147

**Published:** 2019-07-17

**Authors:** Yingmei Jia, Huasong Cai, Meng Wang, Yanji Luo, Ling Xu, Zhi Dong, Xu Yan, Zi-Ping Li, Shi-Ting Feng

**Affiliations:** ^1^Department of Radiology, The First Affiliated Hospital, Sun Yat-Sen University, 58th, The Second Zhongshan Road, Guangzhou, Guangdong 510080, China; ^2^Faculty of Medicine and Dentistry, University of Western Australia, Perth, Australia; ^3^MR Collaboration NE Asia, Siemens Healthcare, Shanghai, China

## Abstract

**Objectives:**

To assess the efficacy of diffusion kurtosis imaging (DKI) and compare DKI-derived parameters with conventional diffusion-weighted imaging (DWI) for distinguishing hepatocellular carcinoma (HCC) from benign hepatic nodules including focal nodular hyperplasia (FNH), hemangioma, and hepatocellular adenoma (HCA).

**Materials and Methods:**

151 patients with 182 hepatic nodules (114 HCCs and 68 benign nodules including 33 FNHs, 29 hemangiomas, and 6 HCAs) were analyzed. Preoperative MRI examinations including DKI (*b* values: 0, 200, 500, 800, 1500, and 2000 sec/mm^2^) were performed, and kurtosis (*K*), diffusivity (*D*), and apparent diffusion coefficient (ADC) were calculated. The efficacy of DKI-derived parameters *K*, *D*, and ADC for distinguishing HCC from these benign nodules was analyzed.

**Results:**

ROC (receiver operating characteristic curve) analysis showed the optimal cutoff values of ADC, *D*, and *K* for identification of these benign nodules, and HCCs were 1.295 (area under the curve (AUC): 0.826; sensitivity 80.6%; specificity 70.8%), 1.787 (AUC: 0.770; sensitivity 83.6%; specificity 59.6%), and 1.002 (AUC: 0.761; sensitivity 65.5%; specificity 79.0%), respectively. Statistically significant differences were found in ADC, *D*, and *K* values between groups of HCC-FNH and HCC-hemangioma (*P* < 0.05). There were significant differences in *K* and ADC values between groups of FNH-hemangioma and HCA-hemangioma (*P* < 0.05), respectively. Using logistic regression analysis, a regression equation was obtained: Logit(*P*)=−1.982*X*_1_+1.385*X*_3_+1.948(*X*_1_: ADC; *X*_3_: *K*), and odds ratios (OR) were 0.138 (95% confidence interval (CI): 0.052, 0.367), and 8.996 (95% CI: 0.970, 16.460), respectively.

**Conclusion:**

Both ADC value and DKI-derived parameters *K* and *D* values have demonstrated a higher preoperative efficacy in distinguishing HCC from FNH, hemangioma, and HCA. No evidence was shown to suggest *D* or *K* value was superior to the ADC value.

## 1. Introduction

Liver nodules are commonly encountered on clinical investigations with a wide differential diagnosis. With the rapid development of imaging modalities and the continuous improvement of their sensitivity, the incidence of liver nodules detection has increased in the recent decades [1]. Liver nodules can be classified according to neoplastic nature of the lesions, including benign or malignant neoplasms as well as tumor-like lesions [2]. Hepatocellular carcinoma (HCC) is the most common cause and is responsible for approximately 90% of primary malignancies in the liver [3–5]. Accurate preoperative diagnosis and identification of HCC are essential in determining the optimal treatment. More and more newer imaging techniques have been developed to improve the preoperative diagnostic accuracy of HCC [6].

Magnetic resonance imaging (MRI) is an important technique used for risk stratification and treatment planning in patients with HCC. Dynamic contrast-enhanced imaging is the most commonly used MRI sequence for identification of benign and malignant liver nodules. However, some HCCs may exhibit similar appearances to other benign hepatic nodules, such as focal nodular hyperplasia (FNH), hemangioma, and hepatocellular adenoma (HCA) [7]. These overlapping radiological characteristics result in variable specificity in HCC detection [8]. The combination of hypointensity on T1-weighted images, hyperintensity on T2-weighted images, and arterial phase enhancement with washout in the portal venous on dynamic gadolinium-enhanced images may be the most common appearance of HCC on MR imaging. However, atypical radiological features may confuse the diagnosis of HCC with other benign hepatic nodules. Small HCCs less than or equal to 15 mm are frequently isointense on both T1- and T2-weighted images and do not demonstrate the typical contrast enhancement during arterial phase images because of its hypovascular structure [9]. Furthermore, the use of gadolinium can be limited in patients with chronic renal failure due to the risk of nephrogenic systemic fibrosis, resulting in a more difficult radiological diagnosis of HCC in this group of patients [10]. Radiological differentiation of HCC from other benign hepatic nodules remains challenging.

As one of the additional nonenhanced MRI methods, diffusion-weighted imaging (DWI) has been proposed to improve the diagnostic specificity of DCE imaging for liver nodules. DWI was demonstrated to be a useful adjunct sequence to DCE imaging, improving diagnostic accuracy of liver nodules compared to DCE imaging alone. Apparent diffusion coefficient (ADC) is obtained by the monoexponential model of conventional DWI with the assumption that water diffusion obeys the Gaussian law [11]. Previous studies have reported DWI has improved the sensitivity and detection rate of HCC when combined with enhanced MRI [12]. However, some reports have also suggested that it may be difficult to differentiate HCC from other solid hepatic lesions like FNH, HCA, and metastasis by DWI due to the overlapping ADC values between HCC and these solid lesions as a result of their increased cellularity [11, 13, 14]. In 2005, Diffusion kurtosis MR imaging (DKI), first proposed by Jensen et al. [15], was described as water diffusion deviates from the Gaussian law due to the complexity of microenvironment, such as cell membranes, vessels, or macromolecules. DKI can provide information about the heterogeneity and irregularity of tissue components. Compared with the ADC value of conventional DWI, the parameters of DKI may have greater potential in reflecting the characteristics of the HCC microstructure [16–18].

The role of applying DKI to improve lesion detection and disease grading has been previously investigated in neurological, renal, prostate, and breast malignancies [19–22]. In terms of its use in HCC detection, Wang et al. [23] showed that higher mean kurtosis values are potential predictors for microvascular invasion (MVI) which is indicative of HCC. Budjan et al. [24] reported that DKI was feasible and enables quantitative differentiation between malignant and benign liver lesions. However, whether DKI is superior to DWI in distinguishing HCC from other liver nodules remains unclear. Thus, in this retrospective study, we aimed to explore the application value of DKI with higher *b* values in differentiating hepatic nodules by calculating and analyzing the correlations between various parameters of conventional DWI and DKI in different hepatic lesions, respectively.

## 2. Materials and Methods

### 2.1. Subjects

The study was approved by the local institute review board. The requirement for informed consent was waived off.

**Figure 1:**
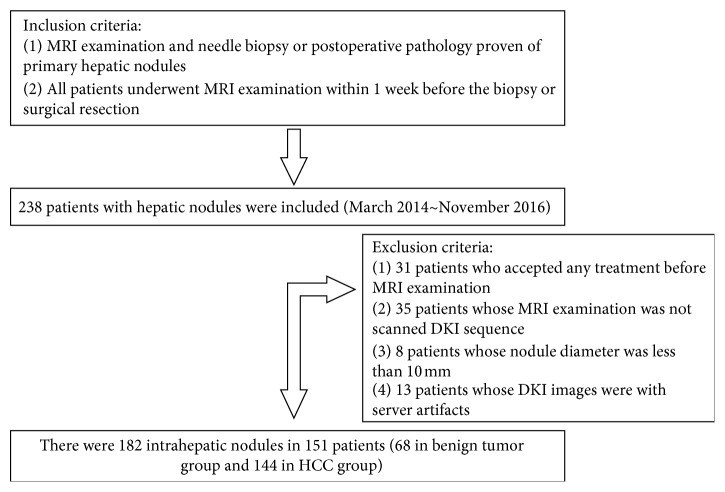
Flowchart of the selection process.

Our study retrospectively collected 238 patients at the First Affiliated Hospital, Sun Yat-Sen University, between March 2013 and November 2016. All patients underwent MRI before the treatment was commenced. 87 patients were excluded in accordance with the following exclusion criteria: (1) patients who have been commenced on any treatment prior to MRI examination (31 cases); (2) patients without DKI sequence performed during their MRI examination (35 cases); (3) patients with lesions less than 10 mm in diameter only (8 cases); (4) patients whose images had severe motion artifacts (13 cases). In the end, 151 patients (119 males and 32 females, with median age of 50 years) were included in the study with 182 hepatic nodules (114 HCC and 68 benign nodules including 33 FNHs, 29 hemangioma, and 6 HCAs) (Figure 1).

All of the HCCs and HCAs were confirmed by surgery or biopsy. FNH and hemangioma were diagnosed on the basis of typical MR imaging features. FNH was diagnosed when all of the following findings were identified in the lesion: (1) isointense or slightly hypointense compared with the hepatic parenchyma on T1-weighted images (T1WI). (2) Isointense or slightly hyperintense on T2-weighted images (T2WI). (3) Homogeneous enhancement during the hepatic arterial phase. (4) Isointense or slightly hyperintense in relation to the adjacent liver parenchyma during hepatic venous and delayed phases. (5) A visible central scar seen as a hyperintense focus on T2WI and as hypointense on unenhanced T1WI, with Gd-EOB-DTPA uptake during the hepatobiliary phase (HBP) [25]. Hemangioma was diagnosed when the typical radiological findings were identified in the lesions, such as high signal intense compared with the liver on T2WI, peripheral globular enhancement, early total enhancement, presence of the fill in the phenomenon, and prolonged enhancement during the equilibrium phase [26].

### 2.2. MRI Protocol

#### 2.2.1. Routine MRI Scan of the Liver

All MRI examinations were performed using a clinical 3T whole-body system (Magnetom Verio, Siemens Healthcare Sector, Erlangen, Germany) with body array coil (3T; 8-channel body matrix coil). All patients were fasted for 4∼6 hours prior to examination and received training regarding breath holding. All images were obtained in supine position.

MRI sequences: Fast low-angle shot (FLASH) T1WI in/out of phase sequence axial imaging; T1WI FLASH with fat suppression (FS) sequence axial imaging; half-Fourier single-shot turbo spin-echo (HASTE) T2WI sequence axial imaging; axial and coronal postcontrast multiphase three-dimensional volume interpolated breath-hold test (3D VIBE) T1WI (FS) sequence (Table 1).

Dynamic contrast-enhanced MR imaging was performed with Gd-EOB-DTPA (Primovist®, 0.1 ml/kg body weight) administered as bolus injection with a flow rate of 1 ml/s. Arterial phase, portal phase, and equilibrium phase images were obtained by performing 3D VIBE T1WI (FS) sequence during suspended respiration at 30–35 seconds, 65–70 seconds, and 100–120 seconds after injection, respectively. Additional HBP images were obtained at 20 minutes after injection.

#### 2.2.2. DKI Sequence

DKI sequences were acquired 10 minutes consistently after contrast media administration. The DKI sequence was scanned by axial free-breathing interleaved multislice DWI after contrast agent administration [27], using a single-shot spin-echo echo planar imaging (SE-EPI) sequence with b values of 0, 200, 500, 800, 1500, and 2000 s/mm^2^ in three orthogonal directions. Fat suppression was achieved by inversion recovery (Table 1).

#### 2.2.3. Image Analysis

Diffusion-weighted imaging and DKI data were analyzed by using in-house prototype software and on the basis of Matlab (MathWorks, Natick, MA). For the DKI model, the multiple b-value data (0–2000 s/mm^2^) were fitted to the following equation: *S*(*b*)=*S*_0_  ·  exp(−*b* · *D*+*b*^2^ · *D*^2^ · *K*/6), in which S(b) represents the signal intensity in arbitrary units and b represents the b value (s/mm^2^), exp represents exponential function, D represents a corrected ADC accounting for non-Gaussian behavior (10^−3^ mm^2^/s), and K represents the apparent kurtosis coefficient.

The ADC map was generated from the same data set (*b* = 0, *b* = 800) based on the monoexponential model *S*(*b*)=*S*_0_  ·  exp(−*b* · ADC) by using in-house prototype software (MathWorks, Natick, MA).

**Figure 2:**
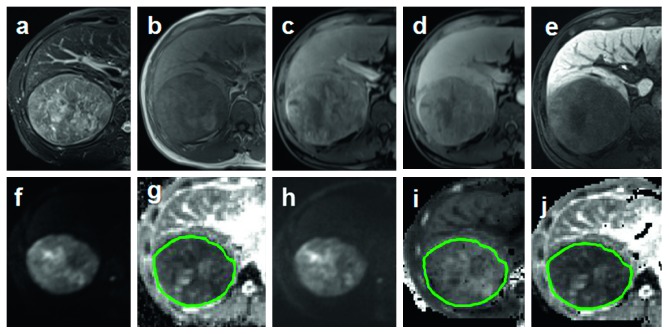
ROI was drawn on the level of maximum transactional diameter of HCC (green circle). (a, b) Axial fat suppress T2WI and T1WI. (c–e) Dynamic enhancement imaging including artery phase, portal phase, and hepatobiliary phase (HBP). (f, g) Axial liver DWI (*b* = 800 s/mm^2^) and ADC maps. (h–j) Axial liver DKI (*b* = 2000 s/mm^2^) and DKI-derived parameters *K* and *D* maps.

**Figure 3:**
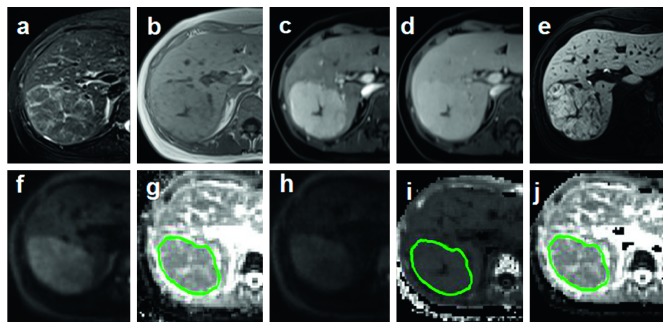
ROI was drawn on the level of maximum transactional diameter of FNH (green circle). (a, b) Axial fat suppress T2WI and T1WI. (c–e) Dynamic enhancement imaging included artery phase, portal phase, and hepatobiliary phase (HBP). (f, g) Axial liver DWI (*b* = 800 s/mm^2^) and ADC maps. (h–j) Axial liver DKI (*b* = 2000 s/mm^2^) and DKI-derived parameters *K* and *D* maps.

**Figure 4:**
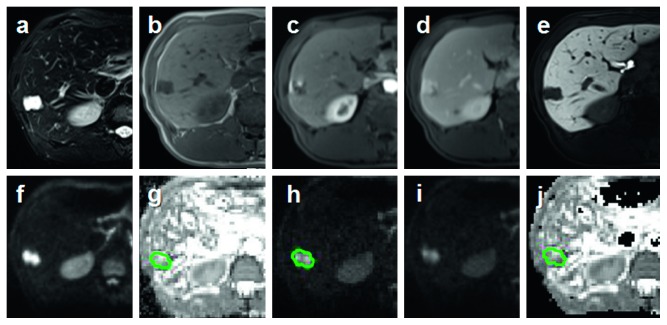
ROI was drawn on the level of maximum transactional diameter of hemangioma (green circle). (a, b) Axial fat suppress T2WI and T1WI. (c–e) Dynamic enhancement imaging included artery phase, portal phase, and hepatobiliary phase (HBP). (f, g) Axial liver DWI (*b* = 800 s/mm^2^) and ADC maps. (h–j) Axial liver DKI (*b* = 2000 s/mm^2^) and DKI-derived parameters *K* and *D* maps.

**Figure 5:**
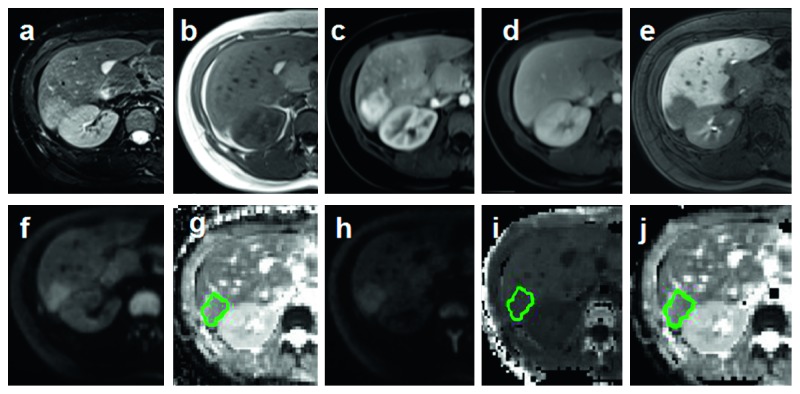
ROI was drawn on the level of maximum transactional diameter of HCA (green circle). (a, b) Axial fat suppress T2WI and T1WI. (c–e) Dynamic enhancement imaging included artery phase, portal phase, and hepatobiliary phase (HBP). (f, g) Axial liver DWI (*b* = 800 s/mm^2^) and ADC maps. (h–j) Axial liver DKI (*b* = 2000 s/mm^2^) and DKI-derived parameters *K* and *D* maps.

All three parameters were independently measured by two experienced gastrointestinal radiologists who were blinded to patients' information. ROIs (regions of interest) were drawn with the same criteria on the level of maximum transactional diameter of the lesion manually, avoiding the obvious areas of hemorrhage, necrosis, and cystic changes (Figures 2–5).

### 2.3. Statistical Analysis

All data were analyzed using the SPSS 20.0 software. Numerical data were expressed as mean ± SD. The Chi-squared test and one-way analysis of variance (ANOVA) were used to compare gender and age distribution amongst the HCC and hepatic benign nodules groups. The independent sample *t*-test or *K* nonparametric test was performed for analysis of ADC, *D*, and *K* between groups of HCC, FNH, and hemangioma.

The ROC (receiver operating characteristic curve) analysis and logistic regression method were used to compare the difference of diagnostic efficacy amongst the parameters. *P* values <0.05 were considered statistically significant.

## 3. Results

182 cases of liver nodules were collected, including 68 cases of hepatic benign nodules group and 144 cases of HCC group. Gender and age distribution of all groups are listed in Table 2. Statistically significant differences in both gender and age were observed amongst the groups (*P* < 0.001).

At the test level of *α* = 0.10, distribution of the parameters of ADC, *D*, and *K* was skewed, so the nonparametric test was chosen. Using the Mann–Whitney *U* test, statistically significant difference with respect to ADC, *D*, and *K* was observed between the groups (Table 3). Furthermore, statistically significant difference with respect to ADC, *D*, and *K* was observed between HCC-to-FNH and HCC-to-hemangioma (*P* < 0.05). A significant difference was observed with respect to *K* between FNH and hemangioma groups (*P*=0.001). There was no significant difference in the spectrum of ADC and *D* between FNH and hemangioma groups (*P* > 0.05). The *P* values of parameters in 3 groups are shown in Table 4.

**Figure 6:**
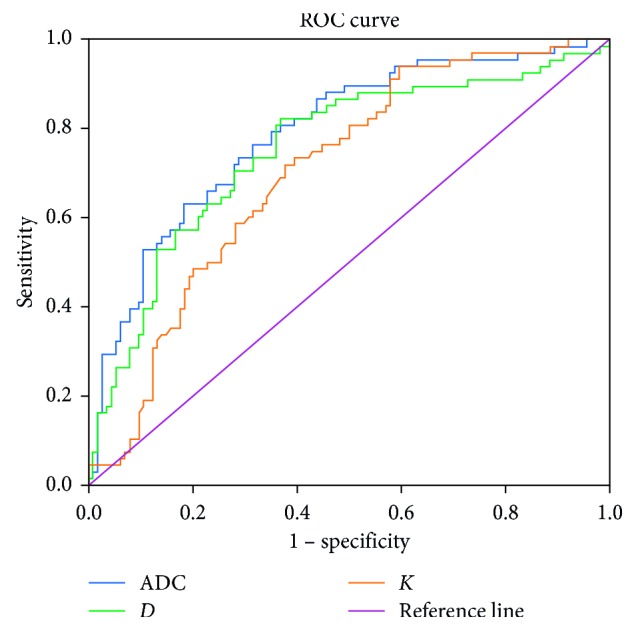
ROC curve analysis shows the accuracy of the *K* value (AUC 0.761; sensitivity 65.5%; specificity 79.0%), *D* value (AUC 0.770; sensitivity 83.6%; specificity 59.6%), and ADC value (AUC 0.826; sensitivity 80.6%; specificity 70.8%) for differentiating between benign nodules and HCC.

On ROC analysis, the optimal cutoff values of ADC, *D*, and *K* for differentiation of HCC from other benign hepatic nodules were 1.295 (area under the curve (AUC): 0.826; sensitivity: 80.6%; specificity: 70.8%), 1.787 (AUC: 0.770; sensitivity: 83.6%; specificity: 59.6%), and 1.002 (AUC: 0.761; sensitivity: 65.5%; specificity: 79.0%), respectively (Figure 6).

Using single factor of the logistic analysis, on the test level of *P* < 0.1, there was statistically significant difference in ADC, *D*, and *K* values (*P* < 0.001), where *X*_1_, *X*_2_, and *X*_3_ are ADC, *D*, and *K*, respectively. Afterwards, multivariate logistic regression analysis was used to obtain the regression equation with the forward stepwise method: Logit(*P*)=−1.982*X*_1_+1.385*X*_3_+1.948, *P* < 0.001, 0.55, and odds ratio values were 0.138 (95% confidential interval: 0.052, 0.367) and 8.996 (95% confidential interval: 0.970, 16.460), respectively. Above all, ADC can be used as a separate protective factor, while the *D* and *K* values cannot be used as a single risk factor.

## 4. Discussion

Our study demonstrated that both ADC and DKI-derived parameters *K* and *D* values can be used to distinguish HCC from those benign hepatic nodules such as FNH, hemangioma, and HCA. The sensitivity of the *D* value was higher than those of ADC and *K* values. We also found that FNH and hemangioma could be distinguished from HCC by using ADC, *D*, and *K* values. However, only the *K* value could differentiate FNH from hemangioma, and the ADC value could differentiate HCA from hemangioma.

As a form of MR imaging, DWI bases on measuring the random Brownian motion of water molecules within a voxel of tissue. Highly cellular tissues or those with cellular swelling exhibit lower diffusion coefficients. As an MRI, it is well known that DWI has been proved to be highly sensitive in detection of HCC. However, in particular when interpreted in combination with enhanced MR, the difference of ADC between the benign liver nodules and HCC remains controversial in the previous studies [28]. Some previous studies have showed that benign lesions had higher ADC values than those of malignant lesions, with a variable degree of overlap. Taouli and Koh [29] summarized different ADC cutoffs for diagnosing malignant hepatic (1.4–1.6 × 10^−3^ mm^2^/sec) with a sensitivity of 74%–100% and specificity of 77%–100%. However, Sutherland et al. [30] and Sandrasegaran et al. [31] found no difference between ADC values of solid benign liver lesions and malignant lesions. One of the main reasons is that ADC values are related to *b* values used for image acquisition, while DWI sequences lack standardization. This may also be the result of the different sample sizes or the different methods used to calculate ADC values.

As an advanced DWI model, DKI has been increasingly implemented for providing more precise information of tissue characteristics than conventional DWI. Compared with conventional DWI assuming the Gaussian behavior of water diffusion, DKI could quantify non-Gaussian behavior of water diffusion, which is closer to the movement and distribution of water molecules within biologic tissues. DKI not only provides a corrected apparent diffusion coefficient (ADC) but also reflects the deviation of tissue diffusion from a Gaussian distribution. On the other hand, DKI is better fitted with image signal attenuation than the monoexponential model, and it can reveal the heterogeneity of the lesions more precisely [32]. In hepatic nodules, the incidence of necrosis, hemorrhage, and cystic degeneration is higher in malignant tumors due to the active degree of proliferation, which makes malignant tumor more heterogeneous in radiological appearance than benign tumor. However, some of the hepatic benign nodules may also demonstrate obvious heterogeneous appearance. Examples include central scar, fiber separation, arteriovenous fistula or inflammation in FNH, small thrombus or cyst degeneration in hepatic cavernous hemangioma, and hemorrhage or steatosis in hepatic adenoma [30, 31, 33]. This finding is consistent with the previously published results evaluating the application of DKI on the diagnostic process for other malignancies. For example, Falk Delgado et al. [34] showed that the *K* value had high diagnostic performance of high- and low-grade glioma because of high heterogeneity in high-grade glioma; In the rectal study, Zhu et al. [35] showed that compared with ADC values, the *K* value was more valuable in the identification of high and low grades of rectal adenocarcinoma and lymph node metastasis.

There are relatively few studies on liver DKI due to low signal-to-noise ratio (SNR) and image artifacts. Goshima et al. showed that the accuracy of *K* value assessment of HCC viability was higher than that of the ADC value [36]. Rosenkrantz et al. [37] used DKI in fresh liver explants and found the DKI model may have added value in HCC characterization in comparison with a standard monoexponential model of DWI [37]. However, our study demonstrated the values of *D* and *K* of DKI are not better than the ADC of conventional DWI for preoperative diagnosis of the hepatic nodules. One possible explanation is that *K* values did not provide stronger correlation with cellularity, it reflected structural heterogeneity among tumors with varying degrees of cellularity, and the main influencing factors are complexity of lesion microstructure. In addition, there is a difference in the heterogeneity between different benign hepatic nodules due to their corresponding unique and complex microstructures [38]. Given the above, the diagnosis efficiency of *K* values may be limited in hepatic malignancy when compared to its application in other organs. In addition, *D* values and ADC values of malignant lesions were significantly lower than those of benign lesions, and *D* values were also shown to have a lower performance than ADC. This can be explained by the fact that the *D* value is a corrected ADC value related to the Gaussian behavior, which is also influenced by *K* values [39, 40].

Different number of *b* values is crucial for reliable DKI data fitting. Previous abdominal DKI studies have indicated that DKI sequence of body needs at least three *b* values (maximum optimal range of 1500∼2000 s/mm^2^), and 3 directions is applied to each *b* value [40]. In our study, DKI was performed with six *b* values ranging from 0 to 2000 s/mm^2^ in three orthogonal directions. Actually, the highest *b* value at 2000 s/mm^2^ in the liver also resulted in lower signal-to-noise ratio (SNR) and faster signal decay of the transverse relaxation [28]. The DKI was acquired with a free breathing sequence allowing greater patient comfort. And, patients must perform respiratory training before MRI examination to minimise breathing artifacts.

In clinical settings, contrast enhancement has been widely used in preoperative qualitative diagnosis of focal lesions in the liver, especially in cases of suspected primary hepatocellular carcinoma. For patients with renal failure and contrast agent contraindications, DWI technology provides a kind of noninvasive examination method that is more conducive to impact on the clinical management [41]. The DKI model, an emerging MRI technology, is more sensitive to tissue microstructural complexity and heterogeneity in high *b* values. However, as mentioned above, at high *b* values, it accelerates the T2 relaxation, which, in turn, reduces the signal-to-noise ratio significantly. In addition, it is difficult to obtain good image quality for high *b* values owing to respiratory motion artifact [42]. All things considered, conventional DWI has a higher significance to be popularized in clinic.

Our study had the following limitations. Firstly, only HCC, HCA, FNH, and hemangioma were enrolled in the study. Other hepatic nodules such as regenerative nodule (RN), dysplastic nodule (DN), intrahepatic cholangiocellular carcinoma, and liver metastases have not been included in this study due to the small sample of cases. Moreover, selective bias could not be avoided in our study as atypical FNH and hemangioma might not have been included in the study. In future studies, we will try to involve more types of hepatic nodules to evaluate the efficacy of DKI for distinguishing HCC from other hepatic nodules. Secondly, in image analysis, the ROIs were only delineated in the maximum transection of the lesion, which may result in selection bias. Thirdly, there were two *b* values less than 200 s/mm^2^ in DWI scanning, which did not comply with the optimal value principle of the biexponential model, and the relationship between liver perfusion parameters and hepatic nodules was not statistically analyzed. Further, despite no significant effect of Gd-EOB-DTPA on DWI had been previously reported [27], the effect of Gd-EOB-DTPA on DKI remains unclear. Consequently, further study is required to explore the DKI acquisition delay after Gd-EOB-DTPA administration.

In conclusion, DKI, based on non-Gaussian diffusion reveals the tissue heterogeneity. *D*, *K*, and ADC parameters of DKI and conventional DWI have higher efficiency for differentiating between benign and malignant hepatic nodules which are useful in the preoperative diagnosis of hepatic nodules. However, the data from this study did not demonstrate the values of *D* and *K* of DKI were better than those of the ADC of conventional DWI in differentiating HCC from benign hepatic nodules.

## Figures and Tables

**Table 1:** Acquisition parameters of the MRI protocol.

Sequence	Imaging direction	Category	TR (ms)	TE (ms)	FOV (mm)	Matrix	FA (°)	Bandwidth	Slice thickness (mm)
T1WI in-phase/out-phase	Axial	FLASH	200	2.2/3.7	328 × 350	192 × 256	65	930/977	6
T1WI FS	Axial	FLASH	235	2.2	328 × 350	240 × 320	70	822	6
Contrast-enhanced									
T1WI	Axial	VIBE	3.3	1.2	328 × 350	128 × 256	13	501	2
T2WI	Axial	FSE	2000	95.0	328 × 350	204 × 256	150	781	6
T2WI FS	Axial	FSE	2000	95.0	328 × 350	204 × 256	150	781	6
DKI	Axial	EPI	4500	66	380 × 380	128 × 128	90	1954	6
HBP T1WI	Axial	VIBE	3.3	1.2	328 × 350	154 × 256	35	501	2

MRI, magnetic resonance imaging; TR, repetition time; TE, echo time; FOV, field of view; T1WI, T1-weighted imaging; T2WI, T2-weighted imaging; FS, fat saturation; DKI, diffusion kurtosis imaging; HBP, hepatobiliary phase; FA, flip angle; FLASH, fast low-angle shot; VIBE, volume interpolated breath-hold examination; HASTE, half-Fourier single-shot turbo spin-echo; EPI, echo planar imaging; FSE, fast spin echo.

**Table 2:** Comparison of gender and age distribution amongst HCC, FNH, and HCA groups.

	FNH	Hemangioma	HCA	HCC	*P* value
Age, yrs, mean ± SD	37.0 ± 1.8	42.5 ± 2.5	41.0 ± 3.8	55.4 ± 1.2	<0.001

Gender					<0.001
Male	14 (50.0)	15 (65.2)	5 (83.3)	85 (90.4)	
Female	14 (50.0)	8 (34.8)	1 (16.7)	9 (9.6)	

Categorical variables are presented as *n* (%). HCC, hepatocellular carcinoma; FNH, focal nodular hyperplasia; HCA, hepatocellular adenoma; SD, standard deviation.

**Table 3:** Comparison of ADC, *D*, and *K* values between hepatic benign nodules and the HCC group.

	Hepatic benign nodules	HCC	*P* values
ADC (×10^−3^ mm^2^/s)	1.691 ± 0.067	1.239 ± 0.039	<0.001^*∗*^
*D* (×10^−3^ mm^2^/s)	2.571 ± 0.116	1.872 ± 0.596	<0.001^*∗*^
*K*	0.856 ± 0.026	1.062 ± 0.318	<0.001^*∗*^

^*∗*^Mann–Whitney *U* test. HCC, hepatocellular carcinoma; *K*, kurtosis; *D*, diffusivity; ADC, apparent diffusion coefficient.

**Table 4:** *P* values for intergroup differences using the Mann–Whitney *U* test.

Groups	Parameters		
	ADC (×10^−3^ mm^2^/s)	*D* (×10^−3^ mm^2^/s)	*K*
HCC-FNH	<0.001	<0.001	0.011
HCC-hemangioma	<0.001	<0.001	<0.001
FNH-hemangioma	0.260	0.985	0.001
HCC-HCA	1.000	1.000	1.000
FNH-HCA	0.085	0.127	1.000
HCA-hemangioma	0.046	1.000	0.086

HCC, hepatocellular carcinoma; FNH, focal nodular hyperplasia; HCA, hepatocellular adenoma; *K*, kurtosis; *D*, diffusivity; ADC, apparent diffusion coefficient.

## Data Availability

The datasets generated during the current study are not publicly available because of patient privacy but are available from the corresponding author on reasonable request.

## Abbreviations

DKI: Diffusion kurtosis imaging
DWI: Diffusion-weighted imaging
HCC: Hepatocellular carcinoma
FNH: Focal nodular hyperplasia
HCA: Hepatocellular adenoma
*K*: Kurtosis
*D*: Diffusivity
ADC: Apparent diffusion coefficient
ROI: Region of interest
AUC: Area under the curve
OR: Odds ratio
PLC: Primary liver cancer
MRI: Magnetic resonance imaging
MVI: Microvascular invasion
T1WI: T1-weighted images
T2WI: T2-weighted images
SE-EPI: Spin-echo echo planar imaging
SNR: Signal-to-noise ratio
HBP: Hepatobiliary phase
FA: Flip angle
RN: Regenerative nodule
DN: Dysplastic nodule.
